# Application of practice-based learning and improvement in standardized training of general practitioners

**DOI:** 10.1186/s12909-024-05195-7

**Published:** 2024-03-01

**Authors:** Bin Yang

**Affiliations:** 1 Health Management Center, Union Hospital, Tongji Medical College, Huazhong University of Science and Technology, 1277 Jiefang Avenue, 430022 Wuhan, Hubei Province P. R. China; 2 Department of General Practice, Union Hospital, Tongji Medical College, Huazhong University of Science and Technology, 1277 Jiefang Avenue, 430022 Wuhan, Hubei Province P. R. China

**Keywords:** practice-based learning and improvement, Resident physician, Standardized training, General practitioner, Competency, Application effect, Education

## Abstract

**Background:**

In the context of standardized training for general practitioners, the emphasis is still primarily on clinical skills, which does not fully encompass the overall development of general practitioners. This study implemented a practice-based learning and improvement (PBLI) project among students and evaluated its effectiveness based on indicators such as learning outcomes, students’ subjective experiences, and annual grades. This study offers recommendations for optimizing general practitioners’ teaching and residential training programs.

**Methods:**

60 residents who participated in the regular training of general practitioners at the First Clinical College of Tongji Medical College of Huazhong University of Science and Technology from January 2019 to January 2022 were selected for this study. They were randomly divided into two groups, the PBLI group, and the control group, using a random number table method. Out of the 60 residents, 31 were assigned to the control group and 29 were assigned to the PBLI group. The participants in the PBLI group received additional PBLI training along with their daily residential training, while the participants in the control group only took part in the latter. The effectiveness of the PBLI program was analyzed by conducting a baseline survey, administering questionnaires, and evaluating examination results.

**Results:**

After implementing the program, the PBLI group scored significantly higher than the control group (*p* < 0.05). Throughout the implementation process, students in the PBLI group expressed high satisfaction with the learning project, particularly with its content and alignment with the training objective. The teacher’s evaluation of the PBLI group students surpassed that of the control group in various areas, including literature retrieval, self-study, courseware development, speech ability, and clinical thinking.

**Conclusions:**

The PBLI program aims to encourage resident-centered study in standardized residency training. This approach is beneficial because it motivates students to engage in active learning and self-reflection, ultimately enhancing the effectiveness of standardized residency training.

## Background

A family doctor is a specialist who provides comprehensive and continuous medical care to individuals of all ages, genders, and conditions [1–3]. Internationally, primary health care systems rely on general practitioners to act as ‘gatekeepers’ and handle 90–95% of patients’ complaints on a long-term basis [4, 5]. This highlights the crucial need for well-trained general practitioners. In China, as the healthcare service model undergoes transformation, general practitioners are assuming an increasingly important role in basic healthcare services [6]. The objective of China’s healthcare reform is to establish an efficient hierarchical diagnosis and treatment system, with a focus on strengthening the role of primary healthcare organizations and general practitioners as the ‘gatekeepers’ of health [7, 8]. There are various types of general-practice residential training teaching models in China. However, most of these models are based on the traditional approach of transferring theoretical knowledge and lack the cultivation of comprehensive abilities such as medical humanities, teamwork, and evidence-based medical concepts. Practice-based learning and improvement (PBLI) is considered a crucial competency throughout a physician’s career, as identified by the Accreditation Council for Graduate Medical Education (ACGME) [9]. While many residency training programs have started recognizing the importance of teaching PBLI programs to residents, there is a lack of studies on PBLI programs specifically designed for general practice residents. This study aims to address this gap by developing a primary care PBLI project that aligns with the existing teaching model and fulfills the requirements of PBLI, which are essential competencies for residents. The project will evaluate the impact of the primary care PBLI project on enhancing physicians’ comprehensive abilities in a general hospital setting.

## Methods

### General information

This study included residency trainees in general practice who underwent standardized training at our general practice specialty base between January 2019 and January 2022. The inclusion criteria were as follows: completion of at least 1 year of residency training, participation in the practice competency-based learning and improvement program in the second year, willingness to cooperate with the study, and provision of informed consent. Trainees who were unable to actively cooperate with the Practice-Based Competency Learning and Improvement Program were excluded. Sixty trainees were enrolled in the study, with 30 trainees enrolled in 2019 and 30 trainees enrolled in 2020. The enrolled trainees were randomly divided into two groups: the PBLI group and the control group. The control group consisted of 31 trainees, with an average age of 30.45 ± 3.07 years. Among them, there were 19 males and 12 females, and 23 trainees passed the Licensing Examination (LE) at the end of the first year of residency training. The PBLI group consisted of 29 trainees, with an average age of 29.55 ± 3.21 years. Among them, there were 15 males and 14 females, and 21 trainees passed the Licensing Examination (LE) at the end of the first year of training. All of the enrolled trainees were socialized trainees without work units or selected trainees from outside units, and they all had a bachelor’s degree. There were no statistically significant differences between the two groups in terms of gender, age, or passing rate of the first year’s medical Licensing Examination (LE) (*P* > 0.05). See Table 1.

**Table 1:** Demographic data in the study population at baseline

	Control group (*n* = 31)	PBLI group (*n* = 29)	x^2^ value/t value	*P* value (95% CI)
Age, yrs	30.45 ± 3.07	29.55 ± 3.21	1.11	0.83
Sex				
Male	19	15	0.559	0.46
Women	12	14		
Pass Rate for LE (%)	23 (74.19%)	21 (72.41%)	0.024	0.88

LE: the Licensing Examination

### Intervention

The general practice residency trainees were categorized based on the residency syllabus requirements. This study focused on the second year of trainee residency training. Trainees in the control group underwent daily general practice residency training. In contrast, trainees in the PBLI group, who were also in their second year of training, participated in an additional learning and improvement program based on practice competence. This program was implemented alongside the regular daily general practice residency training without affecting the original activities, such as difficult case discussions, mini-lectures, teaching rounds, and teaching in the outpatient clinic.

The program followed the ADDIE model (Analysis, Design, Develop, Implement, Evaluate) [10]. In the second year of training, all trainees have become familiar with the training site and determined their learning goals. Conceive and implement PBLI projects through interactive and participatory workshops. Participants in the PBLI group were allowed to form multiple study groups consisting of 3 to 4 participants. Each group was supervised by a general practice faculty member. In accordance with the requirements of the general practice residency training syllabus and in conjunction with their work in residency training bases and primary practice bases, participants reviewed the information on their own. The project settings varied according to different outpatient and inpatient settings (Table 2). Several interactive seminars were organized by the base faculty. The seminars served as a platform for discussions, where the results of the discussions were analyzed to determine the PBLI learning theme. The chosen theme should reflect the characteristics of general medicine and address the necessary and weak learning content of general medicine at present. The general practice faculty played a supportive role throughout the process, ensuring that the trainees’ ideas were appropriate and feasible and that the theme’s content aligned with the actual work of general practitioners. The PBLI theme should be focused on enhancing the competence of general practitioners. Once the theme is selected, a corresponding learning plan should be designed and submitted to the instructor for review. The project lasted for 9 months.

PBLI project implementation method:

1. Step 1, PBLI project design and guidance. For example, if a trainee notices a high number of patients with metabolic syndrome in their primary practice site, they may choose to address the challenge of managing these patients. After group discussions and consultation with the instructor, the trainee decides to take on the theme of ‘Community Management of Metabolic Syndrome’ for their PBLI project and creates an implementation plan.
2. Step 2, all students form a team of 3 to 4 participants and complete at least one Plan-Do-Study-Act (PDSA) cycle as a group, which takes 2 to 3 months. Throughout the project, the group regularly reports progress to their assigned mentor. The first step involves conducting a community survey to determine the epidemiological status of metabolic syndrome in the local population. The second step includes addressing relevant questions such as the diagnostic criteria for metabolic syndrome, goals for blood pressure, blood glucose, blood lipids, and weight management, as well as evidence-based and guideline recommendations. ①Plan: General practitioners announce the primary care PBLI project, and the research team selects one or uses a self-designed case as the report title. ②Do: Based on the selected PBLI project, review information, discuss in groups, and complete a reading report. The content includes the current status of case community management, the latest research results, development trends, existing deficiencies and suggestions for improvement, and the results of the group discussion will be reported at the book report meeting. ③Study: Accept feedback and evaluation from different groups of research subjects and instructors, self-reflect, understand deficiencies and guide self-learning. ④Act: Modify the plan and put forward suggestions for improvement. Enter the next PDSA cycle. Completion of at least 2 PDSA cycles is the requirement for the end of this training. The PBLI project has a set timeframe, and participants regularly report their progress to the instructor. At the end of the project, the group of trainees presents a completion report to all general practice training trainees and faculty. The new program is then implemented, and feedback and evaluation are provided.
3. Step 3: Evaluation of PBLI project training effects. The implementation effects of the PBLI project were evaluated using three dimensions: satisfaction evaluation, objective results, and teacher evaluation.

**Table 2:** PBLI projects implemented:

1. Purpose-oriented treatment of hypertension
2. Antibacterial drug management plan
3. Reduce the readmission rate of patients with normal ejection fraction heart failure
4. Community residents outpatient drug regulation
5. Early outpatient follow-up after discharge of heart failure
6. Community management of metabolic syndrome
7. Reduce the risk of bleeding in patients with coumarin
8. Prevention and discussion of deep venous thrombosis
9. Patient safety inspection and safety culture
10. Improving community management of coronary heart disease
11. Management of chronic kidney disease

### Data collection

The Plan-Do-Study-Act (PDSA) model is used as the learning framework for PBLI. After completing the PBLI project in the second grade, we evaluated the implementation effect using the self-made questionnaires ‘Resident PBLI Ability Training Satisfaction Questionnaire’ and ‘Teacher Satisfaction Evaluation Questionnaire’.

1. General practitioners’ satisfaction evaluation: The Resident PBLI Ability Training Satisfaction Questionnaire was developed based on the PBLI competency training questionnaire from the University of Wisconsin School of Medicine and Public Health. The overall Cronbach’s alpha coefficient of the Index System was 0.982 [16]. A total of 342 questionnaires were distributed, and 319 valid questionnaires were collected, resulting in an effective recovery rate of 93.27%. All items in the evaluation form were rated on a 5-point Likert scale and were completed promptly after the program concluded. The questionnaire included items assessing satisfaction with the content, format, effectiveness, alignment with residency training objectives, and overall satisfaction with the PBLI program. Participants can rate their satisfaction on a scale of 1 to 5, with 1 indicating very dissatisfied, 2 indicating moderately dissatisfied, 3 indicating generally satisfied, 4 indicating moderately satisfied, and 5 indicating very satisfied.
2. Evaluation of all trainees by general practitioners: The Teachers Satisfaction Evaluation Questionnaire is a self-designed questionnaire. A total of 231 questionnaires were distributed, out of which 212 valid questionnaires were collected, resulting in an effective recovery rate of 96.10%. All assessments were performed using a 5-level Likert scoring method. The evaluation encompassed trainees’ competence in “literature research”, “case analysis ability”, “independent learning”, “courseware production skills”, “speaking ability”, and “clinical thinking ability”. Each question offered 5 options:‘very good’, ‘good’, ‘average’, ‘poor’, and ‘very poor’. Any result that could be classified as “very good” or “good” was considered as being part of the satisfaction result, and any other result was rated as a nonsatisfaction result (any score of “average”, “poor” or “very poor”).
3. The annual appraisal performance of the two groups of trainees in general practice specialty residency training was compared.

### Data analysis

Data analysis was conducted using SPSS 27.0 statistical software. The count data are presented as frequencies and percentages, while the measurement data that followed a normal distribution are expressed as the mean ± SD ($\bar x\, \pm \,s$). The chi-square test and *t* test were used to analyze the count and measurement data, respectively. *P*<0.05 was considered statistically significant.

### Ethical considerations

This study received approval from the Medical Ethics Committee of Union Hospital, Wuhan, China. Informed consent was obtained from all participants involved in the study. All methods were conducted in strict adherence to the applicable guidelines and regulations.

## Results

### Self-assessment by participants in the PBLI group

A total of 29 trainees participated in the PBLI group, and 11 PBLI projects were finalized. A total of 319 valid questionnaires were received to evaluate the impact of the PBLI project implementation. Relative to the comparison group, the residents in the PBLI curriculum demonstrated a significant increase in the content of the project with a mean score of 4.64 ± 0.79, the project’s format with a mean score of 3.68 ± 0.81, and the effectiveness of the conducted project with a mean score of 3.95 ± 0.68. They also reported satisfaction with the alignment between the PBLI program and the objectives of residency training with a mean score of 4.35 ± 0.86, as well as overall satisfaction with the PBLI program with a mean score of 4.16 ± 0.78 (Table 3).

**Table 3:** The survey results of PBLI group participants' satisfaction with PBLI program (*n* = 319)

Content	Score ($\bar x\, \pm \,s$)
content of the PBLI project	4.64 ± 0.79
form of the PBLI project	3.68 ± 0.81
results of the PBLI project	3.95 ± 0.68
fit between the PBLI project and the residential training objectives	4.35 ± 0.86
overall satisfaction with the PBLI project	4.16 ± 0.78

### Instructor evaluation of the two groups of participants

The study included 60 participants in both groups, with each participant being evaluated by more than three instructors. A total of 212 valid evaluation surveys were collected, consisting of 102 evaluations for the trainees in the PBLI group and 110 evaluations for the trainees in the control group. The questionnaire survey revealed that the trainees in the PBLI group exhibited significantly better skills than the control group in terms of “literature searching skills” (LSS), “independent learning” (IL), “courseware production skills” (CPS), “speaking ability” (SA), and “clinical thinking ability” (CA), and the difference was significant (*P* < 0.05). However, there was no significant difference in “case analysis ability” (CAA) between the two groups (Table 4).

**Table 4:** Alteration of teachers’ satisfaction with the competence of trainees after PBLI programs

	Control group (*n* = 102)	PBLI group (*n* = 110)	x^2^ value	*P* value
LSS				
satisfaction nonsatisfaction	72	95	5.115	0.02
	30	15		
CAA				
satisfaction nonsatisfaction	81	96	1.698	0.19
	21	14		
IL				
satisfaction nonsatisfaction	66	89	4.07	0.04
	36	21		
CPS				
satisfaction nonsatisfaction	65	90	5.077	0.02
	37	20		
SA				
satisfaction nonsatisfaction	73	97	6.151	0.01
	29	13		
CA				
satisfaction	88	105	4.56	0.03
nonsatisfaction	14	5		

LSS: literature searching skills; CAA: case analysis ability; IL: independent learning; CPS: courseware production skills; SA: speaking ability; CA: clinical thinking ability

### Comparison of annual achievements of the two groups of participants

The PBLI program was completed during the second year of the general practice residency, and an annual assessment was conducted at the end of the year. Comparing the scores from the annual assessment, it was found that the PBLI group scored higher than the control group [(120.35 ± 8.98) points vs. (84.97 ± 12.26) points]. The difference between the two groups was statistically significant (*t* = 12.84, *P* < 0.001).

## Discussion

The quantity, quality, and service level of general practitioners have a direct impact on the quality of healthcare services in China [11]. Even though the utilization rate of family doctors in China is only 6.9% [12], a significant number of residents are in a state of being ‘signed but not contracted’. The main reason for the low rate of effective signing is that primary general practitioners (family doctors) lack the necessary skills to attract residents to prefer primary health care institutions. This study focuses on a problem-oriented and patient-centered primary care PBLI project for general practice residents. The project aims to enhance medical safety, promote health, and improve the job competency of general practice residents. Additionally, it aims to enhance the ability of residents to continuously learn and develop their skills in practice. The project involves various activities such as literature search, independent learning, cultural exchange, and teaching general practice-related knowledge. By enhancing the general medical clinical response ability of general practitioners, the project also fosters the development of self-reflection and independent learning abilities in general practitioners [13, 14]. PBLI is one of the six core competency standards for US residents, developed by the ACGME and other organizations [9]. ACGME subdivides competence into six core competencies: (1) Medical Knowledge; (2) Patient Care; (3) Communication Skills; (4) Practice-based Learning and Improvement; (5) Systems based Practice; (6) Professionalism. This study demonstrates that the PBLI program enhances trainees’ abilities in various aspects of self-learning and self-improvement. These include literature searching ability, independent learning ability, courseware production ability, presentation ability, and embodiment of clinical thinking. Trainees who have participated in the PBLI program maintain their interest in learning for a longer period compared to other trainees [15, 16]. The New Century Medical and Health Talent Cultivation Report, published by the 21st century, emphasizes that the core focus of the medical education model based on job competency is to enhance learning ability and develop problem-solving skills through the application of medical knowledge. The standardized training of residents in the United States focuses on student-centered teaching, which requires clinical teachers to tailor their instruction to meet the needs of students and guide their career development. In contrast, the standardized training of residents in China primarily emphasizes the evaluation of teachers’ teaching and professional abilities, with less attention given to the individual needs of students. This project helps to remedy this gap in GP training.

This study conducted 11 PBL projects and found that the residency trainees were highly satisfied with the format and content of the program. They believed that the implementation of the PBLI program helped them achieve the goals of general practice residency training and expressed their willingness to continue participating in its activities. Feedback from the instructors indicated that the PBLI program enhanced the multifaceted competencies of the residency trainees and addressed gaps in current clinical teaching methods for general practice. The PBLI project aims to transform the teaching mode from traditional preaching training to a practice-based approach. It utilizes the Plan-Do-Study-Act (PDSA) model as a systematic experimental learning framework to enhance the practical abilities of general practitioners. The objective evaluation primarily focuses on the annual performance of all students. Following the training, the annual assessment scores of all trainees were higher compared to those of the control group. This suggests that the PBLI training program can effectively enhance the clinical diagnosis and treatment abilities and performance of general trainees. These findings align with **Xu Jiao**’s research, which concluded that innovative models can improve the clinical diagnosis and treatment capabilities of general medicine residents through self-evaluation and objective testing [17]. The project aims to shift the training mode for recent graduates from a biomedical model to a comprehensive understanding of diseases from biological, psychological, and social perspectives. This transformation aims to improve the post-competency training of general practitioners. This study examines the impact of the PBLI project on the comprehensive abilities of all students, taking into account teacher evaluation. “Literature searching skills”, “case analysis ability” and “independent learning” are important components of the general practitioner’s job competence [18]. The results indicate that participation in the PBLI project leads to improvements in students’ abilities in literature retrieval, independent learning, courseware production, and speaking. These findings highlight the advantages of different traditional teaching models and demonstrate successful teaching outcomes. This aligns with previous research conducted by **Zheng Shuping** on the innovative TEST teaching model, which is based on problem-based learning, group teaching, and other teaching models [19]. It can be seen that the PBLI program is closely integrated with the actual content of primary health services, focuses on solving the difficulties in actual work, and provides a method of self-learning and self-experience for general practice residency trainees, which is conducive to the improvement of the competency of general practice positions.

The subjective survey results of this study indicate that there was no significant improvement in ‘case analysis ability’ after training. The analysis suggests that this may be due to limited opportunities for general trainees to handle complex cases and inadequate training. The patients they encountered were non-standardized, and the resident doctors lacked experience in managing such cases. In **Zhou Lin’s** study [20], the use of standardized patients proved to be more effective in enhancing the clinical diagnosis and treatment abilities of general practitioners in training, particularly in doctor-patient communication skills. To address this issue, it is recommended to select more typical cases for study during the implementation of the PBLI project. Additionally, increasing the exposure of general trainees to standardized patients is necessary to improve their doctor-patient communication skills.

However, it is important to note some limitations of this study. It was conducted in a single center and included only 60 standardized training students of general practice residents, resulting in a small sample size and limited generalizability of the conclusions. In future research, efforts will be made to enhance and promote the development of PBLI projects, improve teacher training, and allocate more spare time for students to participate in PBLI projects. Furthermore, larger-scale research involving multiple centers will be conducted.

## Conclusions

This document discusses the application value of PBLI in standardized training for general practitioners. Students have the freedom to choose their own PBLI topics, search for information, and report on them, which stimulates their enthusiasm for self-directed learning. The results indicate that students who have undergone PBLI training maintain a longer interest in learning compared to other students. The PBLI group also showed higher satisfaction in terms of their ability to retrieve literature, engage in self-learning, produce courseware, express themselves in speech, and think clinically. Feedback from the guidance teacher indicates that the PBLI project has helped develop diverse abilities in residential trainees, which addresses the current shortcomings in general clinical teaching. The study concludes that the implementation of PBLI promotes student-centered learning and improves the effectiveness of resident physician training.

## Data Availability

The datasets analyzed in the current study are available from the corresponding author on reasonable request.
